# Supplementary material for: Hydrogen storage with gravel and pipes in lakes and reservoirs
Source: Nat Commun. 2024 Sep 4;15:7723. doi: 10.1038/s41467-024-52237-1 (PMC11375118; doi:10.1038/s41467-024-52237-1)
Supplement: Supplementary file 1 — Supplementary Information [file 41467_2024_52237_MOESM1_ESM.pdf]

Hydrogen storage with gravel and pipes in lakes and reservoirs

Julian David Hunt[1,2], Andreas Nascimento[3], Oldrich Joel Romero[4], Behnam Zakeri[2,5], Jakub Jurasz[6], Paweł B. Dąbek[6], Tomasz Strzyżewski[7], Bojan Đurin[8], Walter Leal Filho[9], Marcos Aurélio Vasconcelos Freitas[10], Yoshihide Wada[1]

Supplementary Information

Supplementary Table 1

Comparison between oceanic vs. reservoir underwater compressed gas energy storage.

	Ocean	Lake/reservoir
Advantages	- Great depths, result in high storage pressures and lower storage costs.<br>- Colder temperatures in the deep ocean.<br>- Large space availability.<br>- In case of an accident, the gas dissipation will be fast.<br>- Possibility of storing gases from off-shore operations, such as hydrogen production with off-shore wind power.<br>- Explosion hazards in floating storage facilities can be avoided.	- Freshwater increases the lifetime of system components.<br>- No currents at the bottom of the lake or reservoir.<br>- Possibility of storing green hydrogen produced from hydropower, wind, or solar power.<br>- Low risk of sabotage of the tank.<br>- Short distance from the bottom of the reservoir to the land.<br>- Existing infrastructure surrounding hydropower reservoirs.<br>- Explosion hazards in aboveground storage tanks can be avoided.
Disadvantages	- Salinity of the water increases corrosion.<br>- Strong currents in the bottom of the oceans.<br>- Distance from the deep ocean to the coast.<br>- High risk of sabotage of the tank.<br>- Stormy weather can impact construction and operation and increase the	- Relatively low depth, which results in medium storage pressures and higher storage costs.<br>- Appropriate locations to install the storage tanks might be restricted due to the bathymetry of the lake or reservoir.<br>- In case of an accident, the gas dissipation will take longer. Depending on the gas, it

[1] Biological and Environmental Science and Engineering Division, King Abdullah University of Science and Technology, Thuwal, Makkah, Saudi Arabia, julian.hunt@kaust.edu.sa.
[2] International Institute of Applied Systems Analysis (IIASA), Laxenburg, Niedaöstareich, Austria.
[3] Federal University of Itajubá, Itajubá, Minas Gerais, Brazil.
[4] Federal University of Espírito Santo, São Mateus, Espírito Santo, Brazil.
[5] Institute for Data, Energy, and Sustainability (IDEaS), Vienna University of Economics and Business (WU), Vienna, Austria.
[6] Wrocław University of Science and Technology, Wrocław, Województwo dolnośląskie, Poland.
[7] Institute of Meteorology and Water Management, National Research Institute, Warsaw, Masovian, Poland.
[8] Department of Civil Engineering, University North, Koprivnica, Koprivnica-Križevci, Croatia.
[9] Faculty of Life Sciences, Hamburg University of Applied Sciences, Hamburg, Lower Saxony, Germany.
[10] Federal University of Rio de Janeiro, Rio de Janeiro, Rio de Janeiro, Brazil.

	maintenance costs of the system.<br>- High costs of offshore operations.	can have severe impacts on the lake or reservoir environment.

Storing other gases in lakes and reservoirs

Other gases can be stored in the same way. Supplementary Table 2 presents some gases that can be stored in lakes and reservoirs. Methane, ethane, and propane can form hydrides at certain temperatures and pressures. The formation of hydrates will impede the gas from flowing out of the tank. Ethane, propane, and carbon dioxide are stored as a liquid in the tank, significantly reducing the tank volume required to store the same mass when compared to storing these elements in a gaseous state. Gases with high solubility in water cannot be stored using the approach proposed in this paper because a high share of the gas will be lost as the water leaves the tank. Also, toxic gases such as ammonia and carbon monoxide cannot be stored with the proposed method because they will impact aquatic life. For instance, 1 mg L^{-1} of ammonia in water can kill fish at 0.5 mg L^{-1} [1].

Supplementary Table 2

Gases that can be stored in lakes and reservoirs.

Gas	Solubility in water at 0°C (mg kg^{-1})[2]	Toxicity	Phase change pressure at 15°C (bar)[3]	Lake and reservoir storage suitability
Hydrogen	1.9	Not toxic	-	Yes
Helium	1.7	Not toxic	-	Yes
Oxygen	69	Not toxic	-	Yes
Nitrogen	29	Not toxic	-	Yes
Methane	39	Not toxic	-	Yes, if no hydrate formation (i.e., pressure lower than 46.1 bar at 5°C and 81.4 bar at 10°C)[4].
Ethane	132	Not toxic	35.2	Yes, if no hydrate formed (i.e., pressure lower than 5.5 bar at 0°C, 30 bar at 14°C, 195 bar at 17°C and 890 bar at 25°C)[5].
Propane	47	Low toxicity	5.9	Maybe with membrane and small quantities to control toxicity if no hydrate formed (i.e., pressure lower than 1.7 bar at 0°C and 5.4 bar at 5.3°C, at higher temperatures, the pressure increases significantly)[5].
Carbon dioxide	3,350	Medium toxicity (Reduce reservoir pH).	49.5	Maybe with membrane and at small quantities to control reservoir acidity and if no hydrate formed[6].
Carbon monoxide	44	Toxic	-	No, due to high toxicity

Ammonia	900,000	Extremely toxic (1 mg L^{-1} can kill fish)	15.0	No, due to high solubility and toxicity

Hybrid hydrogen and compressed air energy storage

An interesting possibility to increase the energy storage flexibility of hydrogen storage in lakes and reservoirs is to use the tanks to store hydrogen in seasonal cycles. Once the hydrogen is extracted from some of the tanks, these tanks can be used to store compressed air (CAES) and provide energy storage in hourly, daily and weekly cycles. The tanks need to be completely emptied of hydrogen before they can be used to store compressed air, and they would be completely emptied of air before it is used to store hydrogen. Hydrogen and compressed air pipelines would be required to connect the compressors on the land with the tanks at the bottom of the lake or reservoir. Comparing hydrogen and compressed air storage, compressed air stores 1% of the energy stored within the hydrogen. However, if this tank is filled and discharged 200 times a year, it will generate two times more electricity than if the tank was filled with hydrogen once per year. To make the plant a hybrid hydrogen and compressed air energy storage facility, additional compressors/decompressors are required to increase the installed power capacity for the plant and allow the tanks to be filled with and emptied of compressed air in daily and weekly cycles. We suggest using isothermal compressor/decompressors with higher efficiencies than other compressor technologies[7,8]. Supplementary Table 3 presents a comparison of using the storage tanks for hydrogen and compressed air energy storage.

Supplementary Table 3

Comparison between hydrogen and compressed air energy storage.

Plant details	Hydrogen	Compressed air
Number of tanks in the plant	450	
Volume per tank (m^3)	4836	
Pressure (bar)	20.6	
Energy storage per tank (GWh)*	190.6	1.84
Energy storage 100% hydrogen (GWh)	85.8	0
Energy storage 50% hydrogen (GWh)	42.9	0.415
Energy storage 0% hydrogen (GWh)	0	0.830
Seasonal storage cycles in one year	1	-
Daily storage cycles in one year	-	365

Weekly storage cycles in one year	-	52
Daily generation with compressed air in one year (GWh)***	-	151.5
Weekly generation with compressed air in one year (GWh)****	-	21.6
Seasonal fuel cell installed capacity (MW)**	28	-
Daily CAES installed capacity (MW)***	-	115
Weekly CAES installed capacity (MW)****	12	-

* Generation efficiency of 70% with hydrogen and compressed air [8].
** The fuel cell operates at 35% capacity factor.
*** Assuming only 50% of the tanks store compressed air and 15% storage capacity.
****Assuming only 50% of the tanks store compressed air and 20% storage capacity.

Supplementary Information References

1. Joel, O. & Amajuoyi, C. Determination of the Concentration of Ammonia that could have Lethal Effect on Fish Pond, PAKISTAN. *ARPN J. Eng. Appl. Sci.* **6(8)**, (2010).
2. Engineeringtoolbox. Solubility of Gases in Water vs. Temperature. *Engineeringtoolbox* https://www.engineeringtoolbox.com/gases-solubility-water-d_1148.html (2023).
3. Air Liquide. Air Liquide Gas Encyclopedia. *Air Liquide* https://encyclopedia.airliquide.com/ (2023).
4. Matsumoto, R. Methane Hydrates. in (ed. Steele, J. H. B. T.-E. of O. S.) 1745–1757 (Academic Press, 2001). doi:https://doi.org/10.1006/rwos.2001.0042.
5. Aregbe, A. G. A Generalized Correlation for Predicting Ethane, Propane, and Isobutane Hydrates Equilibrium Data in Pure Water and Aqueous Salt Solutions. *Glob. Challenges* **3**, 1800069 (2019).
6. Algaba, J. *et al.* Simulation of the carbon dioxide hydrate-water interfacial energy. *J. Colloid Interface Sci.* **623**, 354–367 (2022).
7. Hunt, J. D. *et al.* Compressed air seesaw energy storage: A solution for long-term electricity storage. *J. Energy Storage* **60**, 106638 (2023).
8. Hunt, J. D. *et al.* Isothermal Deep Ocean Compressed Air Energy Storage: An Affordable Solution for Seasonal Energy Storage. *Energies* vol. 16 at https://doi.org/10.3390/en16073118 (2023).
